# Portal hypertension caused by essential thrombocythemia: A case report

**DOI:** 10.1097/MD.0000000000045855

**Published:** 2025-11-07

**Authors:** Yutao Fang, Kaili Xiang, Yihui Liu, Liyuan Tao, Linlin Shi, Dongya Chen

**Affiliations:** a Department of Gastroenterology, Hangzhou Red Cross Hospital, Hangzhou, Zhejiang, China; b Department of Nuclear Medicine, Hangzhou Cancer Hospital, Hangzhou, Zhejiang, China.

**Keywords:** essential thrombocythemia (ET), myeloproliferative neoplasms (MPNs), non-cirrhotic portal hypertension (NCPH), portal hypertension (PHT), venous thrombosis

## Abstract

**Rationale:**

Portal hypertension (PHT) results from increased intrahepatic vascular resistance and augmented portal venous blood flow. Although cirrhosis remains the predominant etiology of PHT, non-cirrhotic PHT also requires clinical attention. This article reports a rare case of PHT with portal vein thrombosis and gastroesophageal varices secondary to essential thrombocythemia (ET).

**Patient concerns:**

An 82-year-old female patient was admitted due to “hematemesis and melena,” abdominal computed tomography revealed ascites, altered liver morphology, mild splenomegaly and PHT. The patient was admitted with a diagnosis of “decompensated cirrhosis.” She maintained normal liver function over a 6-month follow-up, subsequently, the condition progressed to portal vein thrombosis, leading to gastrointestinal bleeding. Laboratory assessments at this stage revealed normal levels of albumin, liver enzymes, bilirubin, and coagulation factors, with no ascites formation, inconsistent with decompensated cirrhosis.

**Diagnoses:**

ET was diagnosed based on Bone Marrow Aspiration and Biopsy, the JAK2 mutation was identified with a variant allele frequency of 42.2%. PHT and thrombosis in this case were attributed to ET.

**Interventions:**

The patient was treated with hydroxyurea and ruxolitinib, endoscopic injection cyanoacrylate/sclerotherapy and endoscopic variceal ligation were performed.

**Outcomes:**

Post-discharge, oral antitumor agents and aspirin for antiplatelet therapy were prescribed, with outpatient follow-up.

**Lessons:**

ET can also lead to PHT, portal vein thrombosis, and esophageal varices. Clinicians should consider these possibilities when dealing with such patients.

## 1. Introduction

Portal hypertension (PHT) is caused by various reasons increased intrahepatic resistance and portal blood flow, clinical manifestations of splenomegaly, hypersplenism, esophageal and gastric varices and ascites. Cirrhosis is the most common cause of PHT, while PHT is also the most frequent clinical manifestation of cirrhosis, it should be noted that not all cases of PHT are cirrhosis-related. Non-cirrhotic portal hypertension (NCPH) refers to a group of diseases in which patients have obvious manifestations of PHT but no evidence of cirrhosis in clinical biochemistry, imaging or histology. There are obvious differences in etiology, treatment, and prognosis between the 2 diseases. Therefore, attention should be paid to the understanding of this group of diseases.^[1–4]^ Here, we report a case of PHT caused by essential thrombocythemia (ET).

## 2. Case presentation

**Figure 1.**
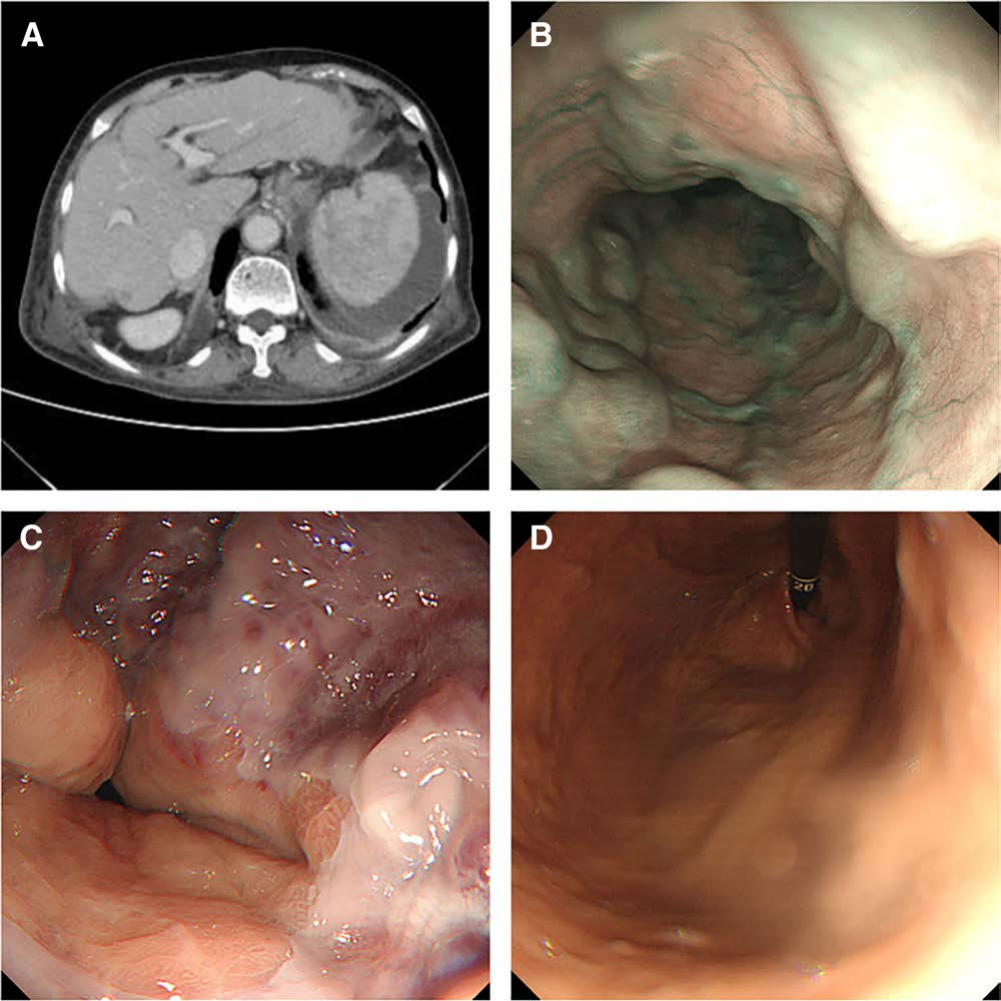
(A) Abdominal CT (2024.03.14) showed ascites, altered liver morphology, mild splenomegaly and portal hypertension. (B) Gastroscopy indicated severe esophageal varices. (C) Red color sign. (D) Image of gastric fundus.

An 82-year-old female patient was admitted due to “hematemesis and melena” on March 13, 2024.The patient vomited approximately 300 mL of dark red blood and had multiple episodes of loose black stools, she had no history of chronic diseases such as hypertension, diabetes, coronary heart disease, kidney disease, or lung disease, and denied any history of hepatitis, schistosomiasis, alcohol consumption, or exposure to toxic substances or medications. She presented with pallor suggestive of anemia with no jaundice in the skin or sclera, no liver palms or spider angiomas, Physical examination of the abdomen revealed no positive findings. Laboratory tests showed: white blood cells 39.0 × 10^9^/L, red blood cells 3.76 × 10^12^/L, hemoglobin 81 g/L, platelets 1986 × 10^9^/L; total bilirubin (TB) 20.90 μmmol/L, albumin 29.5 g/L; prothrombin time (PT) 17.8 seconds, D-dimer 1080.0 μg/L; fecal occult blood test was positive. Hepatitis panel was negative, autoantibodies were negative, and ceruloplasmin was normal. Abdominal CT revealed ascites, altered liver morphology, mild splenomegaly and PHT (Fig. 1A). The patient was admitted with a diagnosis of “decompensated cirrhosis, suspected gastroesophageal variceal bleeding, and thrombocytosis.” Gastroscopy indicated severe esophageal varices (Fig. 1B), with a positive red color sign (Fig. 1C), while no obvious gastric fundal varices were observed (Fig. 1D). Treatment included acid suppression, gastric protection, hemostasis, and fluid replacement for symptomatic management and gastrointestinal bleeding stabilized.

**Figure 2.**
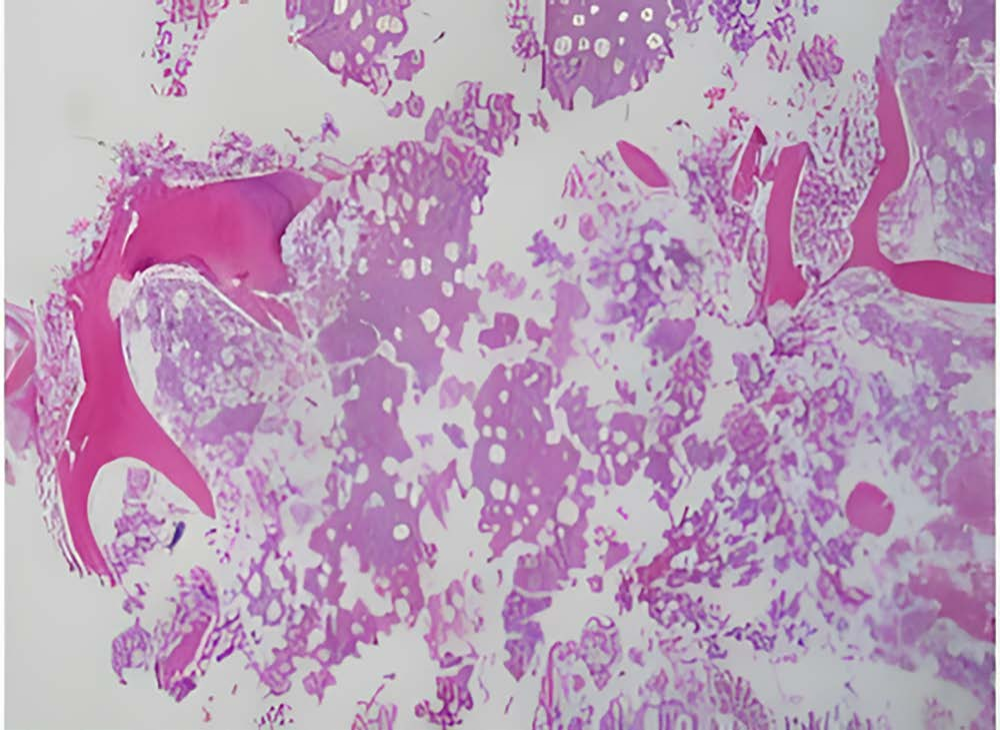
Bone marrow examination indicated an increased number of megakaryocytes and platelet aggregation.

Bone marrow aspiration indicated an increased number of megakaryocytes with good functionality and platelet aggregation (Fig. 2). Flow cytometry did not detect significant immunophenotypic abnormalities associated with acute leukemia, high-risk myelodysplastic syndromes, lymphoma, or myeloma. Biopsy revealed markedly hypercellular marrow with increased megakaryocytes and morphological abnormalities. A JAK2 mutation was identified with a variant allele frequency of 42.2%. The diagnosis was ET, and the patient was treated with hydroxyurea and ruxolitinib. She discharged on 04.24. Post-discharge, oral antitumor agents and aspirin for antiplatelet therapy were prescribed, with outpatient follow-up.

**Figure 3.**
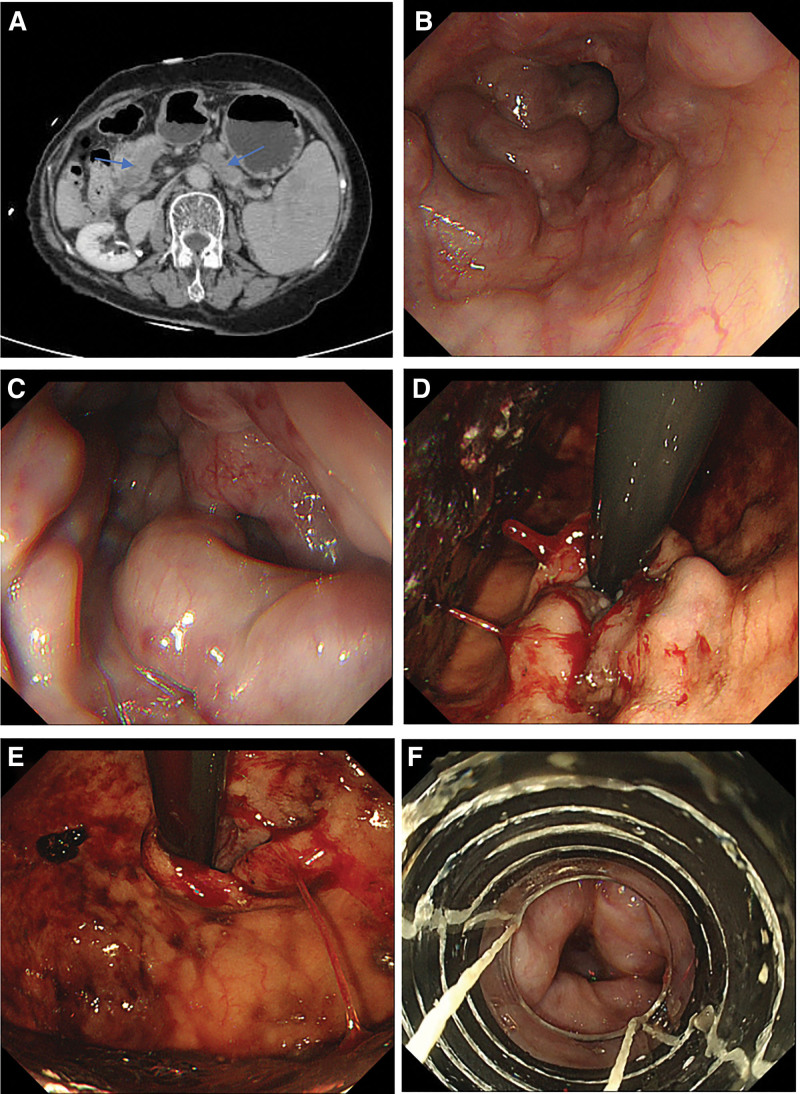
(A) Abdominal CT (2024.11.22) showed dilation of the splenic vein and main portal vein with thrombosis (blue arrow). (B) Gastroscopy indicated severe esophageal varices. (C) Red color sign. (D) Active bleeding sites. (E) Image of gastric fundus: extensive blood staining adherent to the gastric mucosa while no obvious gastric fundal varices were observed. (F) Endoscopic variceal ligation.

The patient was readmitted due to “melena” on November 14, 2024, vital signs are stable. Physical examination reveals no positive findings. There are no manifestations of chronic liver disease. Laboratory tests revealed the following: TB 36.10 μmol/L, albumin 40.8 g/L; (PT) 15.1 seconds, D-dimer 830 μg/L; white blood cells 10.9 × 10^9^/L, red blood cell 3.36 × 10^12^/L, hemoglobin 88 g/L, and platelet 483 × 10^9^/L. CT and ultrasound findings included: dilation of the splenic vein and main portal vein with thrombosis, multiple thrombi in the intrahepatic left and right branches; liver cirrhosis, splenomegaly, and esophageal varices (Fig. 3A). The second endoscopic examination also indicated severe esophageal varices (Fig. 3B) with a positive red color sign (Fig. 3C). Additionally, obvious active bleeding sites were identified (Fig. 3D), accompanied by extensive blood adhesion in the stomach (Fig. 3E), while no significant gastric fundal varices were observed. Endoscopic injection cyanoacrylate/sclerotherapy and Endoscopic variceal ligation were performed (Fig. 3F). The patient’s condition improved with symptomatic treatment, and she were discharged. Regular outpatient follow-up has been maintained to date.

## 3. Discussion and conclusions

NCPH comprises various conditions such as extrahepatic portal vein obstruction (EHPVO), congenital hepatic fibrosis, idiopathic noncirrhotic portal hypertension, hepatic sinusoidal obstruction syndrome, and Budd-Chiari syndrome. EHPVO involves the blockage of blood flow in the extrahepatic portal vein and its branches, leading to prehepatic PHT. In adults, EHPVO is mainly caused by portal venous thrombosis (PVT) and tumor compression, while in children, it is primarily due to congenital developmental anomalies. Myeloproliferative neoplasms (MPNs), such as myelofibrosis, polycythemia vera, ET, and chronic myeloid leukemia, commonly underlie PVT, resulting in the obstruction of blood flow in the main extrahepatic portal vein and its branches, thereby serving as a significant cause of prehepatic PHT.^[5,6]^

ET is a chronic myeloproliferative neoplasm, representing a clonal disorder of hematopoietic stem cells, with an incidence ranging from 1 to 2.5 cases per 100,000 individuals. The condition is distinguished by splenomegaly, thrombocytosis, leukocytosis, and aberrant karyotypes, often manifesting with symptoms like headaches, visual disturbances, and dizziness. Individuals with ET are at an increased risk of both thrombotic and hemorrhagic events.ET is caused by different gene mutations, most common mutation is JAK2V617F, responsible for around 60% of all ET cases and is most likely the main genetic driver of the disease. According to the 2016 WHO diagnostic criteria this patient was diagnosed with ET.^[7,8]^

ET-induced PHT is relatively rare and the mechanisms involved are remain unclear. Currently, the most widely accepted hypotheses include splenomegaly increases portal blood flow, contributing to hyperdynamic circulation. Extramedullary hematopoiesis and sinusoidal fibrosis cause intrahepatic obstruction, elevating portal vascular resistance. In patients with JAK2 mutations, elevated platelet-derived adhesive factors (e.g., P-selectin), reduced activity of anticoagulant proteins (Protein C and S), and neutrophil-mediated reactive oxygen species production promote endothelial injury and thrombosis, ultimately leading to PHT. The mutation in ET prolonging the interaction between peripheral blood and sinusoidal endothelium, resulting in splanchnic venous thrombosis.^[9–14]^

For this patient, it was not accurate enough to diagnose cirrhosis because the phenotypic features of the cirrhosis seen on CT could be related to PHT caused by ET. In addition, intrahepatic or extrahepatic PVT caused by ET may be associated with PHT. Imaging results from the patient’s initial hospitalization indicated changes in hepatic echogenicity and morphological irregularities, indicative of cirrhosis. Despite this, the patient did not have any preexisting chronic liver conditions and did not exhibit common clinical signs associated with liver disease, such as characteristic facial features, jaundice, palmar erythema, or spider angiomas. Although a liver biopsy was not conducted, over a 6-month follow-up, the patient maintained normal liver function (Table 1). Subsequently, the condition progressed to PVT, leading to gastrointestinal bleeding necessitating hospitalization. Laboratory assessments at this stage revealed normal levels of albumin, liver enzymes, bilirubin, and coagulation factors, with no ascites formation, inconsistent with decompensated cirrhosis. Therefore, PHT and thrombosis in this case were attributed to ET. The cirrhotic features likely arose from PHT and PVT rather than the conventional post-injury cirrhosis commonly encountered in clinical settings.

**Table 1:** The patient’s liver function during the half-year follow-up period.

Date	Alb (g/L)	TB (µmol/L)	Pt (s)
March 13, 2024	29.5	20.9	19.9
April 23, 2024	29.5	10.8	15.5
April 30, 2024	34.1	27.6	14.4
May 6, 2024	33.5	10.4	13.7
May 20, 2024	35.2	7.8	13.5
June 28, 2024	36.2	12	15
July 3, 2024	39.5	10.8	14.5
November 14, 2024	40.7	36.1	15.1
November 23, 2024	34.2	10.8	14.9
December 4, 2024	35.7	15.5	13.7
December 9, 2024	33.9	11.8	14.1

Alb = albumin, PT = prothrombin time, TB = total bilirubin.

MPNs can also lead to PHT, PVT, and esophagogastric varices. Clinicians should consider these possibilities when dealing with such patients. It is essential to emphasize thorough history-taking, physical examination, and differential diagnosis during the diagnostic and treatment process. Avoiding cognitive biases and premature diagnostic closure is crucial to ensure accurate diagnosis and the selection of appropriate therapeutic strategies.

## Author contributions

**Writing—original draft:** Yutao Fang, Kaili Xiang.

**Writing—review & editing:** Yihui Liu, Liyuan Tao, Linlin Shi, Dongya Chen.
